# The new indication of TEVAR for uncomplicated type B aortic dissection

**DOI:** 10.1097/MD.0000000000003919

**Published:** 2016-06-24

**Authors:** Chao Song, Qingsheng Lu, Jian Zhou, Guanyu Yu, Xiang Feng, Zhiqing Zhao, Junmin Bao, Rui Feng, Zaiping Jing

**Affiliations:** Department of Vascular Surgery, Changhai Hospital, Second Military Medical University, Shanghai, P.R. China.

**Keywords:** aortic dissection, endovascular repair, follow-up

## Abstract

The classical therapeutic indication for type B aortic dissection is based on either medication or open surgery; medication therapy is recommended for relatively stable uncomplicated type B aortic dissection. With improvements in endovascular repair and the potential risk of disease progression, it is now necessary to evaluate the requirement for revision of the therapeutic choice of uncomplicated type B aortic dissection based on morphological features and time window. Data from 252 patients diagnosed as uncomplicated type B aortic dissection from 1992 to 2015 were analyzed retrospectively. Among these cases, 117 patients received medication therapy and 135 patients underwent endovascular repair. The 60-month survival rate in the endovascular group was higher than that in the medication group (92.3% vs 67.6%). According to the morphological evaluation, visceral artery involvement and false/true lumen ratios over 0.7 were strong risk factors for medical treatment alone. Increased surgical time and blood loss were found in patients treated in the chronic phase, compared with those who underwent endovascular repair within 14 days of the onset of symptoms. With improvements in aortic remodeling techniques, endovascular repair has been shown to improve long-term survival rates of patients with uncomplicated aortic dissection. Considering the potential risk of death, we recommend that patients with visceral artery involvement and a false/true lumen ratio over 0.7 should receive endovascular repair aggressively. Furthermore, delayed endovascular repair in the chronic phase does not improve the long-term outcome of uncomplicated type B aortic dissection.

## 1 Introduction

Aggressive surveillance and careful control of blood pressure, which are recommended as classical standards for treatment of uncomplicated type B aortic dissection (uTBAD), seem to be safe; however, the long-term results are less than ideal,^[1,2]^ and a considerable proportion of long-term mortality can be attributed to aneurysmal degeneration and aortic rupture. Consequently, this raises the question of whether endovascular repair is a better option for uTBAD with specific morphological features.

Clinical trials of thoracic endovascular aortic repair (TEVAR) for aortic remodeling have shown favorable outcomes at the 1-year follow-up, and improved aortic-related mortality was confirmed in longer-term follow-up.^[3–5]^ Considering that one-third of patients who receive medication therapy remain healthy after 60 months of follow-up and the massive financial cost of endovascular repair, it is necessary to further evaluate risk factors that influence the outcomes of uTBAD in patients, especially in China, which is the largest developing country in the world with an incomplete medical insurance system.^[6]^

In this study, we retrospectively analyzed the long-term results of our initial 20-year endovascular repair experience of treating patients with uTBAD. We focused on morphological risk assessment and determining the effects of the time interval between symptom onset and endovascular repair on long-term outcomes.

## 2 Methods

### 2.1 Patient selection

We retrospectively studied patients with uTBAD treated at the Department of Vascular Surgery in Shanghai Hospital, from 1992 to 2015. Patients were identified by discharge diagnosis based upon confirmatory imaging, which was defined as any spontaneously occurring nontraumatic dissection involving the descending aorta. A total of 252 patients were included in our analysis according to specific inclusion and exclusion criteria:

The following inclusion criteria were applied: (1) no evidence of ongoing intractable chest pain at the time of admission; (2) no evidence of rupture or impending rupture at the time of admission; (3) no evidence of refractory hypertension at the time of admission; (4) no evidence of unstable hemodynamics at the time of admission; (5) no evidence of symptomatic end-organ ischemia (clinical symptoms or laboratory test) at the time of admission.

The following exclusion criteria were also applied: (1) positive pregnancy test at the time of admission; and (2) complete false lumen thrombosis at the time of admission.

Informed consent to follow-up was established for all patients when they were admitted. The Institutional Review Board approved this retrospective research. After achieving blood pressure and heart rate control and being informed of the characteristics of each therapy, patients selected the final treatment plan. Patients enrolled into endovascular treatment signed a consent form approved by our Institutional Review Board.

### 2.2 Medical therapy

All the patients were treated medically at the time of diagnosis confirmation, regardless of any differences in subsequent management. The initial priority was reduction of systolic blood pressure to 120 mm Hg, with renal function monitoring; this was achieved by intravenous Urapidil and beta-blocking agents. In addition, heart rate was maintained below 60 bpm.

When blood pressure and heart rate control had been achieved, oral antihypertensive medication was started in the medication group (n = 117 patients). Medication was administered either alone or in combination to maintain systolic blood pressure below 140 mm Hg. Beta-blockers were administered routinely unless contraindicated, although the selection was left to the discretion of each clinician.

### 2.3 Endovascular repair

The first case of endovascular repair of aortic dissection in China was performed at our center in 1999.^[7]^ Since then, 135 uTBAD patients (enrolled in this study) have undergone endovascular repair after initial medical therapy. Of these cases, 56 were admitted in the acute phase within 14 days after the onset of illness, whereas 43 patients in the subacute phase (within 6 weeks of onset) and 36 chronic patients were included in the endovascular group. Surgical complications included cerebrovascular ischemia, abdominal distension, and procedure-related events.

Technical results were reviewed by the authors, and the details of endovascular repair were recorded for further study. Major stent-graft was identified as the one used to seal the primary tear.

Lumbar drainage was used selectively in 2 patients (1.5%). No complications were related to lumbar drain placement. The drain was clamped after 24 hours of surveillance without any neurologic complications, and was removed after an additional period of 6 to 8 hours. Oral antihypertensive medication was also introduced before the patient was discharged to maintain blood pressure and heart rate during the follow-up.

### 2.4 Follow-up

The follow-up was started on the day the index discharge was determined. Patients were followed until death or until the study end date (September 2015). All-cause mortality was adopted as the primary endpoint to ensure comparability with previous studies of type B aortic dissection.^[1,8]^ Adverse outcomes were divided into overall death (sudden unexplained death and aortic rupture, stroke, myocardial infarction, cardiac tamponade, peripheral artery disease, chronic obstructive pulmonary disease, pulmonary embolism, renal failure, gastrointestinal bleeding, accident, and cancer), adverse events (aortic-related, branch artery occlusion, limited physical activity, gastrointestinal bleeding, impaired renal function, and procedure-related), and readmission (medical treatment, open surgery, and endovascular repair). All types of complications were regarded as secondary endpoints.

### 2.5 Morphologic evaluation

All morphological evaluations were performed by radiologists. Computed tomographic angiography (CTA) was recommended at 3 and 6 months, and annually thereafter. Patients outside Shanghai had their scans performed locally, and these data were then forwarded for evaluation by clinicians at Department of Vascular Surgery in Shanghai Hospital. Some patients were lost to follow-up time and some died during the process; therefore, the number of patients decreased with the duration of time. CTA was used to evaluate the entire aorta and iliac arteries, and 3-dimensional reconstruction was obtained using Aquarius (TeraRecon, Foster City, CA). The number of aortic tears and visceral artery involvement were determined on admission. The minimal true lumen diameter of the aorta and the diameter of the false lumen at the same level were compared between the initial and follow-up CT images. The status of the false lumen on all the available images was collected. Complete thrombosis of the false lumen was defined as absence of blood flow in any portion of the false lumen; otherwise, it was considered to be patent false lumen.

### 2.6 Statistical analysis

In this study, means (±standard deviation [SD]), medians, and ranges were used to describe continuous variables, and categorical variables were expressed as percentages. Clinical variables were compared between the medication and endovascular repair groups. Categorical variables were analyzed using Fisher exact test or chi-square test, whereas continuous variables were analyzed by unpaired *t* test or 1-way analysis of variance (ANOVA) test. Univariate associations between all clinical variables were calculated by Cox regression analysis. Multivariate Cox regression analysis was then applied to all variables that had at least marginal univariate predictive value (*P* < 0.10). All variables were simultaneously adjusted in a single step. Hazard ratio (HR) and 95% confidence interval (CI) were calculated. Survival and time-to-event curves were calculated by the Kaplan–Meier method and compared by log-rank test. A *P* value <0.05 was considered to indicate statistical significance. All analyses were performed using SPSS for Windows (SPSS Inc., Version 14.0, Chicago, IL).

## 3 Results

### 3.1 Patient characteristic

As outlined in Table 1, there were no significant differences with regard to age, sex, body mass index, and pre-existing comorbidities between the medication and endovascular repair groups. Hypertension was the most common comorbidity affecting both groups, which confirmed the necessity of antihypertensive medications. Thoracic and abdominal pain was commonly observed, whereas 4 patients experienced transient lower-extremity pain and 3 reported dizziness at the time of onset. Another 14 patients were diagnosed by routine examination.

**Table 1:**
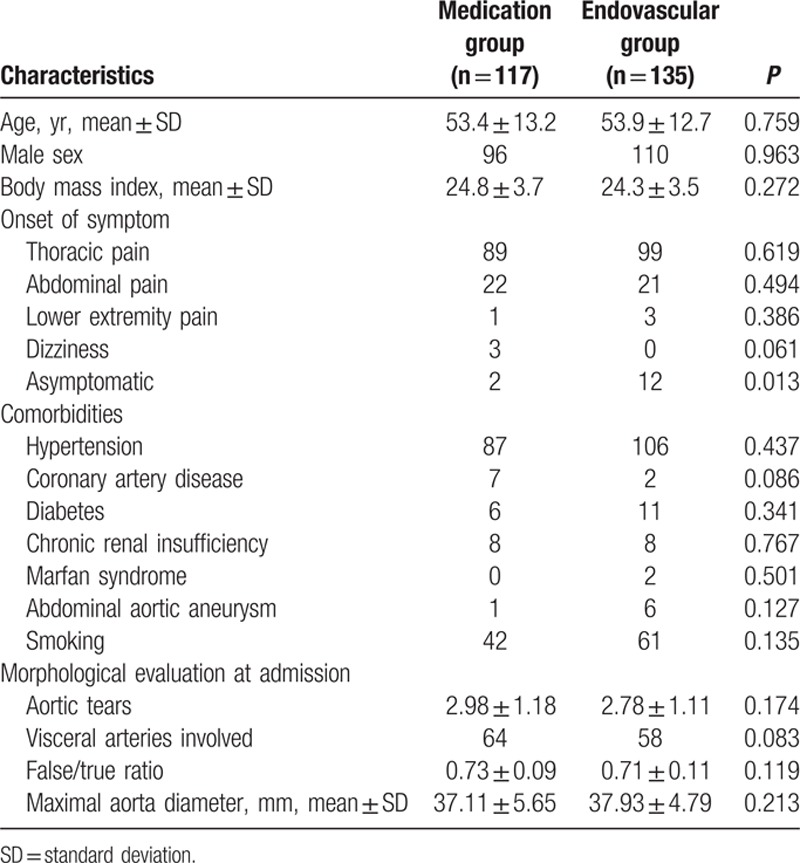
Patient characteristics.

Morphological characteristics were evenly distributed. There were 2.98 ± 1.18 aortic tears in the medication group and 2.78 ± 1.11 in the endovascular repair group. The average false/true (F/T) lumen ratio was 0.73 ± 0.09 in the medication group and 0.71 ± 0.11 in the endovascular repair group. Although visceral arteries were involved in some cases, the dissections in this study were located mainly in the thoracic aorta, which might account for the low number of tears found in our patient cohort.

### 3.2 In-hospital assessment

Similar to other types of open surgery, the durations of the intensive care unit (ICU) stay and hospital stay of patients in the endovascular repair group were significantly different from those of the medication group (Table 2). After 1.2 days of ICU management in the endovascular repair group, the patients were discharged within 20 days.

**Table 2:**
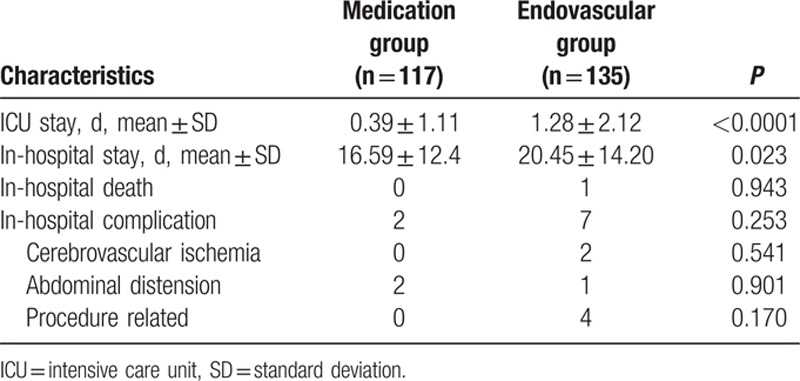
In-hospital assessment.

The in-hospital mortality rates in this patient series was 0% in the medication group and 0.7% in the endovascular repair group. These rates are lower than those (7%–20%) reported in literature,^[9,10]^ possibly due to the hemodynamic stability of uncomplicated patients.

Three patients suffered from cerebrovascular ischemia in endovascular repair group, which might be attributable to the catheterization of the aortic arch during the procedure. Left vertebral artery occlusion was found in 1 patient immediately after stent-graft placement, and although an emergency left carotid-vertebral artery bypass was performed, the patient died 12 days later. Minor stroke occurred in the other 2 patients during hospitalization; these patients were discharged without any sequelae after effective management. No paraplegia occurred during hospitalization in either of the groups.

Three patients in both groups complained of abdominal distension during hospitalization; these patients recovered after the introduction of vasodilators. This type of complication might be attributed to transient dynamic obstruction of visceral arteries.

Despite the risk of procedure-related retrograde type A dissection secondary to endovascular repair, no such catastrophic complications occurred during hospitalization in our patient cohort. One patient suffered from inferior epigastric artery bleeding 1 day after surgery, and emergent endovascular repair was performed successfully to seal the tear. The cause of this complication might be related to the limited flexibility of the delivery system that produced forced wall stress in the external iliac artery leading to intimal injury. Fat liquefaction occurred as a complication in another 3 patients in the endovascular repair group; these patients received daily wound dressing.

### 3.3 Long-term outcome

The mean follow-up in the medication group was 58.4 months and 49.2 months in the endovascular repair group.

**Figure 1:**
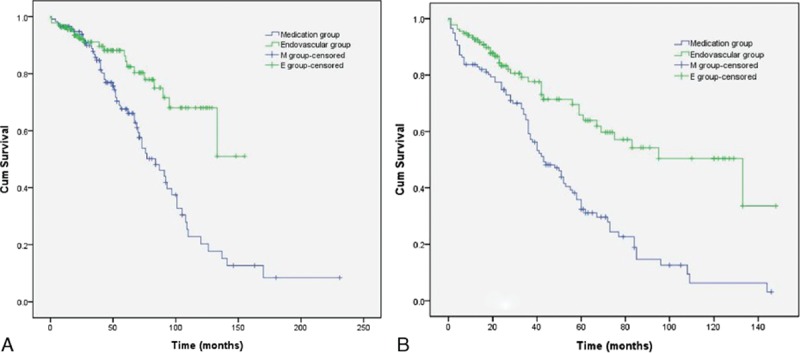
Long-term survival analysis of uTBAD patients. A, Cumulative survival rate in both groups. Statistically significant difference was revealed between curves (log-rank test *P* = 0.002). B, Kaplan–Meier analysis of cumulative freedom from death and adverse events with significant differences between groups (log-rank test *P* = 0.046). uTBAD = uncomplicated type B aortic dissection.

Figure 1 shows the long-term outcome in terms of survival rate and event-free survival. The 24-month survival rates in the 2 groups were equivalent (medication 94.8% vs endovascular repair 96.3%). The 60-month survival rate in the endovascular group was 25% higher than that in the medication group (medication 67.6% vs endovascular repair 92.3%), and the 120-month survival rate in the medication group decreased to 20.3% compared with 68% in the endovascular group.

As summarized in Table 3, the rates of sudden unexplained death and aortic rupture in the medication group were significantly higher than those in the endovascular group (medication 36 vs endovascular repair 11). All types of adverse events were recorded during follow-up; 37 patients in the medication group experienced aneurysmal expansion of the aorta or dissection extension greater than 55 mm, whereas only 4 cases were reported in the endovascular repair group. All these patients received surgical reconstruction or endovascular repair to prevent aortic rupture. Two cases of stroke caused by unilateral carotid dissection were diagnosed in the endovascular repair group, with a greater prevalence of neurological events during long-term follow-up considered unlikely. In the endovascular repair group, we observed permanent paraplegia in 4 (2.9%) patients, and renal function impairment was confirmed in 10 patients, although further evaluation demonstrated the involvement of renal arteries without any signs of ischemia. Nine cases of type I endoleak occurred after discharge, yielding a procedure-related events rate of 81%; these were eliminated by secondary interventions. A nonsignificant retrograde flow without any expansion of false lumen was detected as type II endoleak, and these patients were readmitted for observation with hypotensive medical therapy. In the medication group, 43 patients were readmitted for secondary treatment compared with 24 in the endovascular repair group.

**Table 3:**
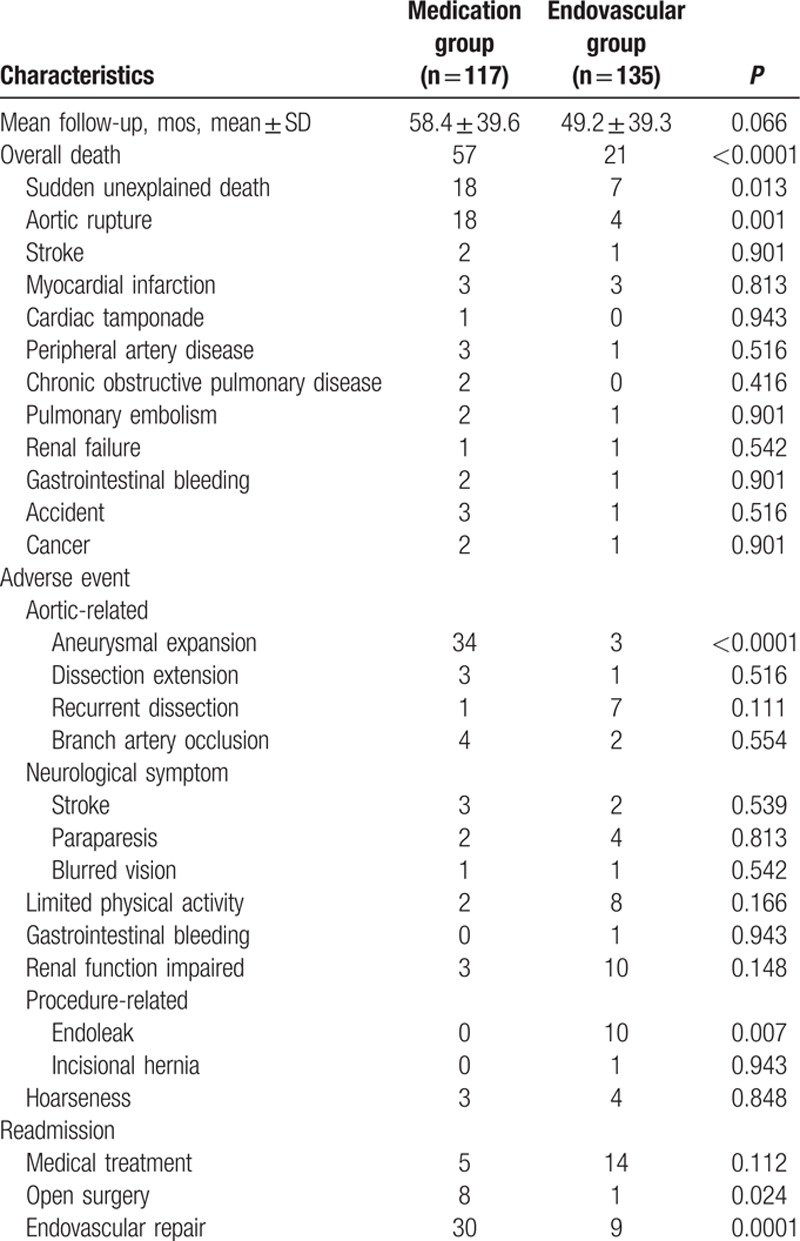
Long-term evaluation of clinical characteristics.

All the images available were collected for morphological evaluation (Table 4). Compared with previous images collected at discharge, there were significant differences in visceral artery involvement between the 2 groups, which suggested successful prevention of longitudinal progression of the aortic dissection in the endovascular repair group. Furthermore, significant improvements in the minimal true lumen diameter were observed only in the endovascular repair group. Evaluation of the status of false lumen thrombosis showed that complete thrombosis was more common in the endovascular repair group.

**Table 4:**
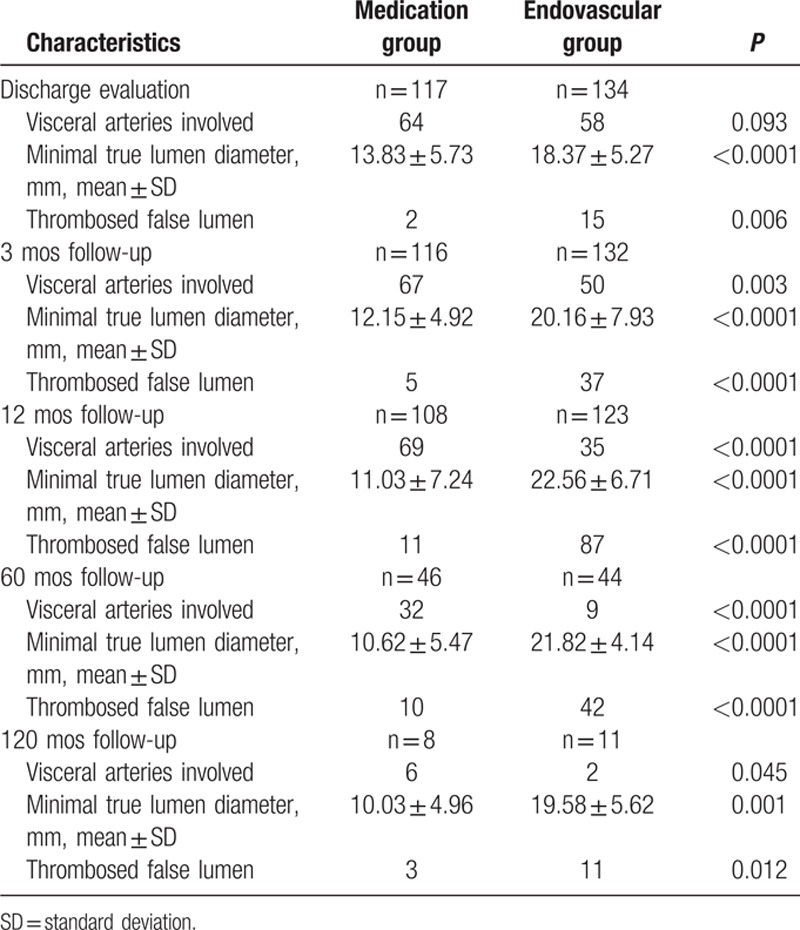
Long-term evaluation of morphological characteristics.

### 3.4 Risk factor analysis

Cox regression analysis predicting postdischarge death is shown in Table 5. Endovascular repair (HR 0.399, 95% CI 0.241–0.660, *P* = 0.000), in-hospital stay (HR 0.978, 95% CI 0.961–0.995, *P* = 0.014), and minimal true lumen diameter (per 1 mm increment, HR 0.914, 95% CI 0.872–0.958, *P* = 0.000) were identified as significant predictors of increased survival. Visceral artery involvement (HR 5.365, 95% CI 2.831–10.167, *P* = 0.000), number of aortic tears (HR 1.634, 95% CI 1.357–1.968, *P* = 0.000), and F/T lumen ratio over 0.7 (HR 4.914, 95% CI 3.157–9.456, *P* = 0.000) were strongly predictive of postdischarge death.

**Table 5:**
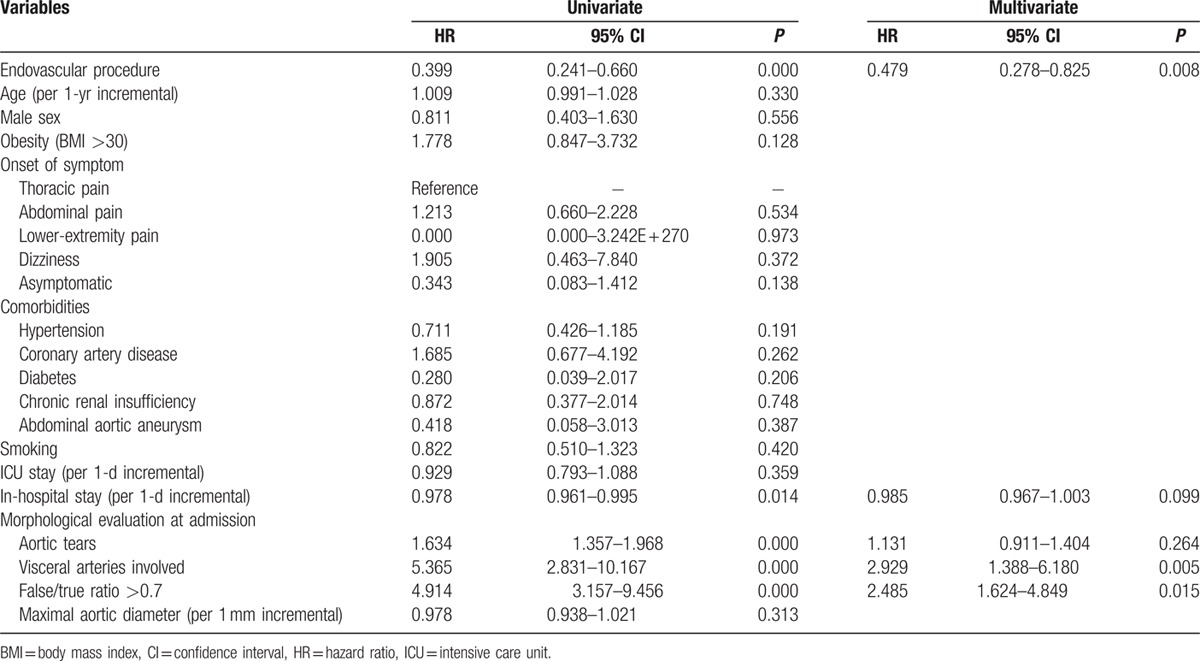
Cox regression analysis predicting all-cause mortality.

Multivariable Cox regression analysis of the all-cause survival rate (HR 0.479, 95% CI 0.278–0.825, *P* = 0.008) confirmed that endovascular repair is a protective factor in long-term survival of uTBAD patients. Visceral artery involvement and F/T lumen ratio over 0.7 were predictive of worse results in the long-term follow-up.

### 3.5 Procedural characteristics evaluation

**Figure 2:**
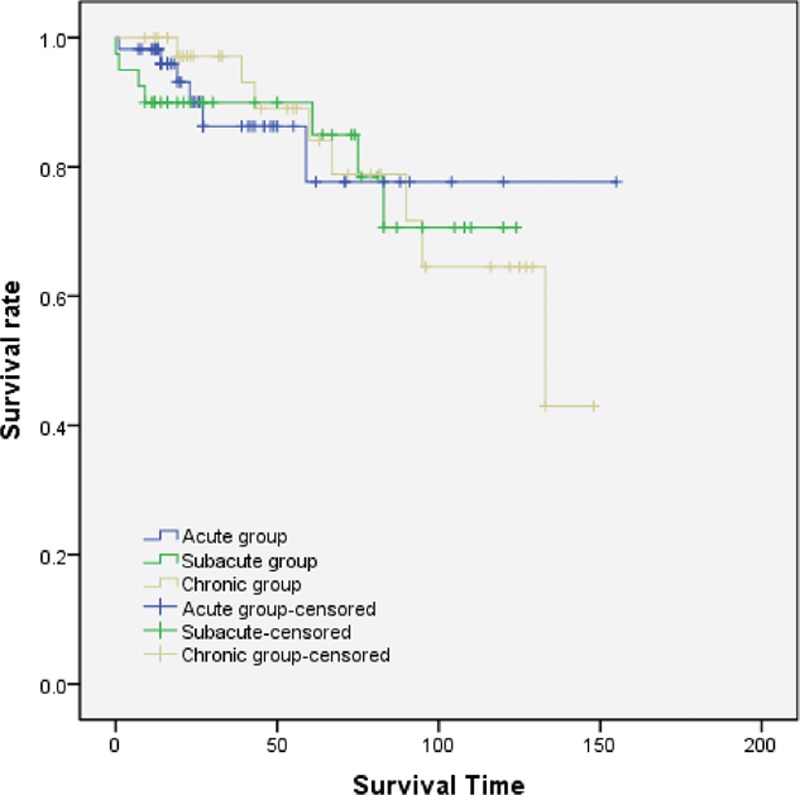
Kaplan–Meier survival analysis stratified by different time interval. The time interval does not affect long-term survival rate of these 3 subgroups (log-rank test *P* = 0.974).

Patients in the endovascular repair group were subdivided into 3 subgroups based on time interval between symptom onset and intervention (acute group <2 weeks, chronic group >6 weeks, and subacute group). Primary closure of the entry tear was achieved in 100% of our patients. Figure 2 shows the all-cause mortality in the 3 subgroups, with no statistically significant difference revealed between the curves.

Procedural details were described in Table 6. Intervention time and blood loss increased significantly in the chronic group compared with the acute and subacute groups. In total, 96.4% patients in the acute phase received endovascular repair via the femoral approach compared with 79% and 77% in the subacute and chronic groups, respectively. General rather than spinal or local anesthesia was more frequently used in the subacute and chronic groups. We also demonstrated that the longer major stent-graft was applied within the first 14 days. There were no differences in the length of ICU and in-hospital stays among the 3 groups.

**Table 6:**
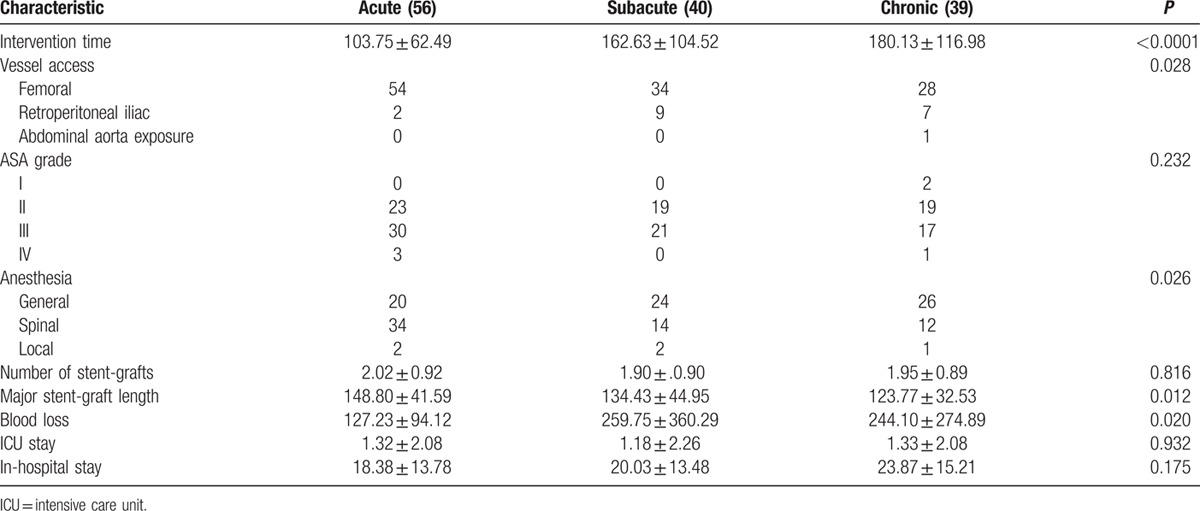
Comparison of procedural characteristics based on time interval.

## 4 Discussion

Although we were unable to identify the exact cause of sudden death due to the lack of autopsy in the medication group, most patients presented with severe pain before death, indicating that dissection rupture might be the cause. Increased diameter of the patent false lumen has been demonstrated to be a significant independent predictor of dissection rupture and aneurysmal degeneration, with the growth rate of the chronically dissected aorta estimated to range from 0.1 to 0.74 cm per year depending on both the initial aortic diameter and the state of hypertension.^[11–17]^ Rudenick et al^[18]^ demonstrated the influence of tear configuration on the false and true lumen hemodynamics in the presence of an adequate outflow at the distal part of the dissection flap, thus, validating distal outflow as a risk factor of progressive dilation. Although lowering outflow with decreased blood pressure may be valuable in such patients, aneurysmal dilatation of the aorta is confirmed at follow-up in more than 20% of medically managed patients, which is consistent with our observation (29%). The therapeutic result of endovascular repair is similar to that of surgical obliteration of the entry tear because the stent-graft can eliminate the proximal tear and redirect blood flow exclusively into the true lumen with diminished outflow. In contrast to the medication group with persistent tears, only 4 patients in endovascular repair group experienced aneurysmal dilation or dissection extension in our study.

In addition to the potential risk of false lumen dilation, tears in the proximal descending aorta with antegrade flow through the false lumen are more likely to be associated with chronic false lumen patency than distal tears with retrograde flow, which is a significant predictor of late mortality and adverse events.^[19–21]^ In addition, similar to the mechanism by which intraluminal thrombosis perturbs the structural integrity and stability of the vessel wall, several previous studies have indicated that partial thrombosis of the false lumen is an independent predictor of mortality.^[22]^ According to these findings, only complete thrombosis of the false lumen is implicated as a potential protective factor of long-term outcomes, and in our study, endovascular treatment of uTBAD was also favored by the 121 cases of complete thrombosis, as compared with 36 cases in the medication group.

The average maximal aortic diameter of patients enrolled in the present study was less than 40 mm, which seems to be safe according to previous studies.^[1,23–26]^ However, true lumen compensation, or even collapse, was detected in the CT scans during follow-up, resulting in a poor prognosis. These findings led us to evaluate the role of F/T lumen ratio in ensuring end-organ perfusion. Although there was no significant difference in the average F/T lumen ratio between the 2 groups at the time of admission, the direct flow was restored in the endovascular repair group with re-expansion of the true lumen, whereas the true lumen was diminished in the medication group at discharge. Multivariable Cox regression analysis demonstrated that increasing F/T lumen ratio had a critical influence on the poor prognosis of patients with uTBAD in our study.

Symptomatic end-organ ischemia has been demonstrated to decrease the long-term prognosis of type B aortic dissection; however, the definition is based mainly on clinical symptoms or laboratory tests. Among the uTBAD patients in the current study, a number of cases of true lumen compression and intimal flap without any symptoms or positive laboratory tests were detected at admission. Evaluation of these patients confirmed that visceral artery involvement is detrimental to long-term survival of uTBAD patients. Consequently, prompt endovascular repair is recommended in patients with visceral artery involvement documented by CT scans, even in the absence of ischemic symptoms at the time of admission.

Aortic tears are another important morphological characteristic of aortic dissection, with unstable patent false lumen correlated with large proximal tears.^[25,26]^ Therefore, we further analyzed the relationship between the number of aortic tears and long-term outcome. Due to closure of the proximal tear in the endovascular repair group, the relationship was evaluated after modification. We reviewed the database to ensure that all the proximal tears sealed by stent-grafts in the endovascular repair group were excluded. In contrast to previous studies, increasing tear numbers did not affect the long-term adverse events of uTBAD patients in terms of the potential aneurysmal dilation according to the multivariable analysis.^[27]^ Although this phenomenon might be explained by the lower number of tears in uTBAD patients than in complicated patients, we consider this finding to be additional evidence of proximal tear closure by stent-graft despite sustained distal retrograde inflow.

Although there is no doubt that patients should receive aggressive medical therapy on admission, the possibility of additional benefits from delayed endovascular therapy remains unclear. To clarify the appropriate time window for uTBAD, we divided the endovascular repair group into 3 subgroups based on the time of application.

Previous studies suggested that the reduction in the stability of the intimal flap in the acute phase is an important determinant of worse survival; therefore, patients should not undergo rapid intervention, because of the weakness of the adventitia and dissection flap and vulnerability to injury induced by stent-graft placement.^[28,29]^

Other studies revealed that impeded stent-graft expansion due to the thick and fibrotic intimal flap is detected more frequently in the chronic phase.^[28]^ Stenosis, tortuosity, or severe calcifications of the iliac axis are also more common in chronic patients, leading to a dangerous shift in the insertion point from the common femoral artery to the common iliac artery or even the abdominal aorta. This change is associated with an increased reintervention rate, extended intervention time, and blood loss.^[30]^ In the present study, 96.4% (54/56) of cases in the acute phase underwent endovascular repair via the common femoral artery compared with 71.8% (28/39) in the chronic group. Furthermore, the intervention time and blood loss were nearly doubled in chronic patients.

Although we failed to confirm that the time interval between symptom onset and intervention is a predictor of survival rate, the low incidence of mortality and morbidity in uTBAD patients may be explained by the evaluation of emergency cases that are mainly complicated reported previously. The increasing intervention time, blood loss, and unconventional insertion point strongly indicate that the difficulty of endovascular repair increases with time. Moreover, the F/T lumen ratio and visceral artery involvement were demonstrated to correlate with intimal flap mobility. Considering that these indexes were ameliorated with aggressive stent-graft placement, performing TEVAR within 6 weeks from symptom onset, with intimal flap stabilization, might improve aortic remodeling and resolution of the dissection. However, emergency endovascular repair should be arranged to avoid the potential risk of paraplegia.

### 4.1 Study limitation

Although the patient number is fairly large and follow-up period is relatively long, this single-center, retrospective study design poses a risk for patient selection bias. The update of both technical expertise and stent-graft design improve during this period, which might influence the result.

## 5 Conclusions

With the improvement of skills and equipment, endovascular repair seems to be a better option for specific group of TBAD patients, with better aortic remodeling. The decreasing F/T lumen ratio and visceral artery involvement along with the classical morphological characteristics (aortic diameter and patent false lumen) predict worse long-term outcome of uTBAD. Accordingly, the proper therapeutic time window of endovascular repair in uTBAD patients should be comprehensive.
